# Successful bridging disconnected pancreatic duct syndrome complicated by plastic stent-induced bowel perforation

**DOI:** 10.1055/a-2584-1811

**Published:** 2025-05-26

**Authors:** Guoming Zhang, Miaomiao Ma, Yi-Fan Wang, Ruiguang Ma, Haiou Li, Zhen Li, Ning Zhong

**Affiliations:** 1 91623 Department of Gastroenterology, Qilu Hospital of Shandong University, Jinan, Shandong, China; 2 Shandong Provincial Clinical Research Center for Digestive Disease, Jinan, Shandong, China; 3 Department of Gastroenterology, The Affiliated Hospital of Qingdao University, Qingdao, Shandong, China; 4 Department of Radiology, Qilu Hospital of Shandong University; Qilu Medical Imaging Institute of Shandong University, Jinan, Shandong, China

A 40-year-old woman with recurrent pancreatitis presented to our institution. She underwent seven endoscopic debridements of a pancreatic pseudocyst with necrosis after an EUS-guided drainage procedure. Since the infiltrates have migrated and encapsulated around the right para-colonic groove, a transnasal tube was inserted for drainage, and subsequently cut by endoscopic scissors to convert to internal gastric drainage, after which she was discharged without symptoms ( Fig. 1 ).

**Fig. 1:**
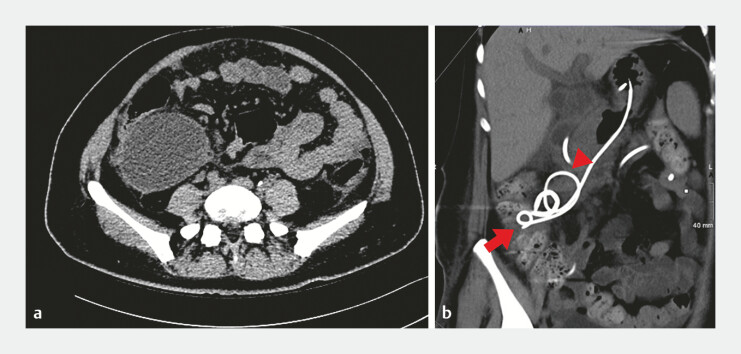
**a** Pancreatitis with pancreatic pseudocyst extension to the right lower pelvic cavity. **b** 3D reconstruction of the internal gastric drainage tube pigtail is intimately connected to the right hemicolon (arrow), and the retroflex segment is adjacent to the duodenum (Triangle).

Following an 8-month gap, she returned with recurrent abdominal pain. Computed tomography (CT) revealed that the stent's distal curved end had entered the colonic cavity. Furthermore, gastroscopy showed that part of the tube had perforated the duodenal wall. We extracted the tube successfully with foreign body forceps, noting its distal end was feces-adhered. Luckily, the duodenal mucosa only showed congestion without bleeding ( Fig. 2 ). Her symptoms were resolved, confirming the tube as the cause rather than suspected recurrent pancreatitis, and the colonic fistula healed spontaneously.

**Fig. 2:**
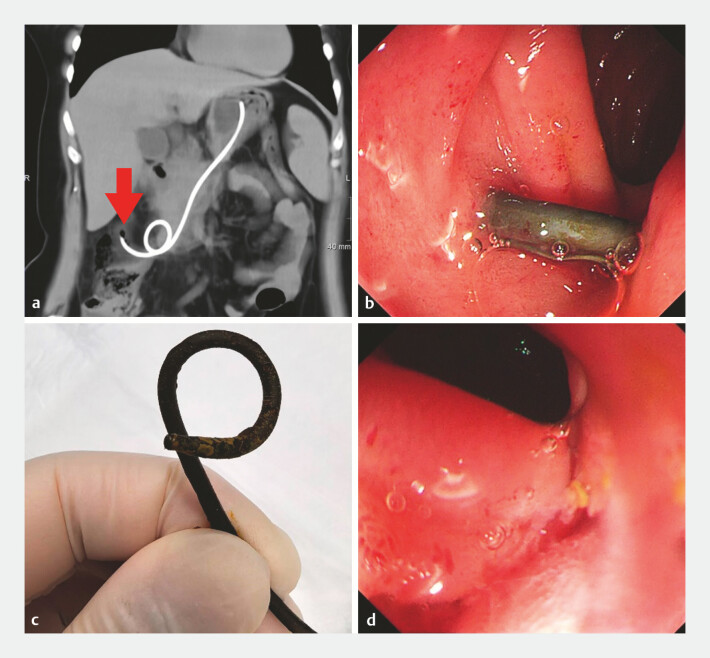
**a** 3D reconstruction of CT revealed the stent's distal curved end had entered the colonic cavity (arrow). **b** The drainage tube was seen to pass through the descending part of the duodenum. **c** Yellowish fecal attachment is seen at the distal end of the drainage tube (Pigtail end). **d** The duodenum showed mucosal congestion without bleeding. Abbreviation: CT, computed tomography.

Another 10 months later, she returned with abdominal pain again. EUS and endoscopic retrograde cholangiopancreatography (ERCP) confirmed disconnected pancreatic duct syndrome (DPDS). However, the guidewire could not pass through the disconnected pancreatic duct (DPD) even with the guidance of pancreatoscopy. Thus, we immediately performed EUS-guided pancreatic duct drainage (EUS-PD) and tried to maneuver the guidewire toward the duodenal side. Unfortunately, the guidewire kept rolling over and failed to bridge the two ends of the DPD. So, we left a 7Fr plastic drainage tube between the cyst and the gastric cavity. After 2 weeks, the cyst almost disappeared, and we successfully bridged the DPD via ERCP ( Video 1 ).

Long-term stent in place can cause duodenal perforations with colonic fistulas. Integrating EUS-guided pancreatic duct drainage before endoscopic retrograde cholangiopancreatography (ERCP) successfully bridged the disconnected pancreatic duct.Video 1

Previous data showed that long-term use of plastic stents effectively managed DPDS without severe adverse events 1 2 . However, this case alerts the potential risk of GI fistula and recurrence of DPDS-related symptoms. We provided a simple way to address such complications and ultimately successfully bridged the DPD.

Endoscopy_UCTN_Code_CPL_1AL_2AD

## Notes

Endoscopy E-Videos https://eref.thieme.de/e-videos
*E-Videos* is an open access online section of the journal *Endoscopy* , reporting on interesting cases and new techniques in gastroenterological endoscopy. All papers include a high-quality video and are published with a Creative Commons CC-BY license. Endoscopy E-Videos qualify for HINARI discounts and waivers and eligibility is automatically checked during the submission process. We grant 100% waivers to articles whose corresponding authors are based in Group A countries and 50% waivers to those who are based in Group B countries as classified by Research4Life (see: https://www.research4life.org/access/eligibility/ ). This section has its own submission website at https://mc.manuscriptcentral.com/e-videos .
